# Sickness-related absenteeism and risk factors associated among flower farm industry workers in Bishoftu town, Southeast Ethiopia, 2018: a cross-sectional study

**DOI:** 10.1186/s13104-019-4223-2

**Published:** 2019-03-29

**Authors:** Tesfaye Hambisa Mekonnen, Soresa Kaba Lamessa, Sintayehu Daba Wami

**Affiliations:** 1 Department of Environmental and Occupational Health and Safety, Institute of Public Health, College of Medicine and Health Sciences, University of Gondar, P.O. Box 196, Gondar, Ethiopia; 2 Labour and Social Affairs Office, Oromia Regional State, Bishoftu Town, Ethiopia

**Keywords:** Sickness absenteeism, Flower farm industry, Prevalence, Predictors, Ethiopia

## Abstract

**Objective:**

The objective of this study was to investigate prevalence and factors affecting sickness absenteeism among flower farm industry workers in Bishoftu town, Ethiopia. A workplace-based cross-sectional study was conducted from March to April 2018. A sample of 444 participants were included using a stratified sampling technique. We performed binary logistic regression analysis to identify factors associated with sickness absenteeism.

**Results:**

The entire sampled workers (N = 444) were interviewed. Of the respondents, 55.6% (N = 247) were females. The mean age was 24.2 (SD ± 6.6) years. About 54.5% (N = 242) [95% CI (50.2, 59.0)] of the participants indicated that they had experienced sickness absence of at least 3 consecutive working days in the past 12 months. A total of 1357 days were lost with an average duration of 5.6 days per worker per year. Female sex [AOR: 2.63; 95% CI (1.723, 4.036)], sickness presenteeism [AOR: 3.15; 95% CI (2.026, 4.904)], job dissatisfaction [AOR: 1.60; 95% CI (1.047, 2.462)] and drinking alcohol [AOR: 1.64; 95% CI (1.023, 2.621)] were associated factors. Sickness absenteeism had been found common in this study. Employers and policy designers need to formulate preventive schemes focusing on gender difference, job satisfaction, and the concomitant tackling of sickness absenteeism and presenteeism.

## Introduction

Sickness absenteeism (SA) is a phenomenon that entails a work disability arising from illness or injury [1, 2]. Absenteeism due to sickness is the major occupational health problems representing substantial costs to workers, employers, and government [3–5]. A recent report (2017) of the International Labor Organization (ILO) showed that more than 340 million occupational accidents and diseases occur on the job every year, of which 50 to 60% of these accidents and diseases cause employees away from work for at least four working days [6]. In the UK, there were about 23.3 million days lost due to work-related ill health, while 4.1 million due to workplace injuries in the period of 2014–2015 years [7]. In the sub-Saharan Africa, each year, 54,000 workers die and 42 million work-related accidents take place resulting at least 3 days’ absence from work [8]. Absenteeism arising from various health conditions is a valuable measure of workers’ health status and capacity to perform and therefore, is an important public health concerns worldwide [5, 9–11]. Moreover, recent prospective study exhibited that sickness absence is a risk marker for all-cause mortality [12].

Manifestation of sickness arising from ill health often varies between different working groups. For instance, it has been shown that 3% of employees absent from work daily in Europe [2]. A study conducted in India showed that about 66.9% of the workers were absent due to health related conditions [13]. A report from Estonia revealed that 8.4% of employees miss work because of conditions related to health problems [14]. An investigation from Brazil described that the prevalence of SA was 31.5% [15]. The studies conducted among agricultural workers in Nigeria [16, 17], India [11], and Ethiopia demonstrated that the prevalence of sickness absenteeism has been found in ranges of 15 to 58.8%.

Several studies explored that multiple predictors induce employees’ absenteeism related to sickness. Socio-demographic characteristics, including sex [18], age of the workers [18, 19], marital status [20, 21], educational level [22, 23], and workplace factors, like periodic medical checkup and working hours, shift work [24], employment type/permanent versus temporary/[25, 26] and work experience importantly increase the risks of sickness absenteeism. Moreover, it has been reported that psychosocial factors, like occupational stress [25, 27], and job satisfaction [14, 25, 28, 29] and behavioral factors, including smoking [30] and alcohol consumption [27, 31] were the factors that predispose employees’ absent from work due to sickness.

In Ethiopia, agricultural sectors, particularly, flower farm industries are rapidly growing, making Ethiopia the 5th largest non-European Union (EU) exporter to the EU cut-flower market and the 2nd largest (after Kenya) flower exporter from Africa [32]. Over 50,000 (70% females) citizens have got job opportunities [32]. However, health and safety protection program is often disregarded and the majority of workers employed in these sectors often suffer from work-related illnesses and injuries. These problems interface with other non-communicable and endemic communicable diseases in the country that persuade to the likely occurrences of employees’ absence from their regular job duties, resulting additional burdens to the public health efforts in terms of healthcare related expenditures. The objective of the current study was, therefore, to explore magnitude and factors affecting sickness absenteeism among flower farm industry workers in Bishoftu town, Ethiopia.

## Main text

### Methods

A workplace-based cross-sectional study was conducted from March to April 2018. The study was conducted in Bishoftu town, Eastern Ethiopia. Bishoftu town is one of the industrial zones in the Eastern part of Oromia regional state, 47 km far from Addis Ababa, the capital of Ethiopia. During the data collection period, there were 12 flower farm industries in the town, employing more than 7330 workers.

#### Populations and sample size

Employees in the flower farm industries in Bishoftu town were the source population. Randomly selected workers whose work activities had direct connection with production department (green house, packing house, spray, cold room, and maintenance room) and had worked for at least 12 months prior to the investigation were included. The required sample was calculated using Epi Info version 7, assuming a proportion (p) of 58.8% [33], and 95% confidence interval (CI) with 80% power and 4% margin of error. With 10% grant for non response rate, we included a final 444 eligible participants. The stratified sampling technique was employed to select the samples, considering that SA varies in each department. A proportional allocation was used to take samples from each stratum.

#### Data collection procedures

The structured questionnaire was interviewer-administered for data collection. The questionnaire was adopted from the literature [33]. We assessed workplace stress by 8-items workplace stress scale questionnaire [34]. The instrument is measured based on 5-Likert scale responses (never = 1, rarely = 2, sometimes = 3, often = 4 and very often = 5) and added together to attain a summary score of 40. A final score was categorized in to two with a score of less than 21 = 0 (not stressed) and a score of 21 and more = 1 (stressed). Perceived job satisfaction was evaluated using 10-items generic job satisfaction scale developed by Scott Macdonald and Peter Macintyre [35]. This is also measured on 5-likert scale responses (from strongly disagree = 1 to strongly agree = 5). The response scales are added and summarized out of 50. We dichotomized the scores in to a score of less than 32 = 0 (dissatisfied) and a score of 32 and above = 1 (satisfied) with their current jobs. Both instruments have been used in previous study conducted in Ethiopia [33]. Moreover, we tested the reliabilities of both tools and found a reliable Cronbach’s alphas (0.813 for the 8-items workplace stress and 0.797 for the 10-items generic job satisfaction scale). Other detail information was obtained regarding sex, age, religion, marital status, educational status, monthly salary, experience, and employment type (permanent and temporary). Data on workplace factors, like pre-employment and periodic employment medical examination (Yes/No), overtime (Yes/No), shift work (day and night)/ (Yes/No), working hours per week (≤ 48 h and > 48 h), attendance incentives (Yes/No), work department (green house, packing house, spray, cold room, and maintenance room) were also assessed.

#### Measurement of sickness absenteeism

Data on sickness absenteeism was extracted from industry registered sick-leave certificates presented to industries in the previous 12 months. In Ethiopia, a worker who is absent from work on grounds of sickness (excluding maternity leave) should present a valid medical certificate from government recognized health facilities the day following his absence. Out of 7330 employees working in the 12 selected flower farm industries, 1291 reported their truancy due to illness in the past 12 months. Of these, 80 cases not presented their medical certificates from government recognized health facilities and were excluded. The remaining 1211 cases of sick-listed and certified by recognized medical organizations were included. These cases were entered into Epi info software from which 444 samples were drawn for a final inclusion. The identity number of the workers was served as a code to cross-link to the response categories of a sickness absence spells (0 = less than 3 days and 1 = 3 days and more) collected by face-to-face interview questions. These categories were recoded in to ‘No’ for 0 = less than 3 days and ‘Yes’ for 1 = 3 days and more to measure sickness absence spells. Those which correctly cross-linked to the indicated medical conditions and sick-leave certificate records of 3 days and more in the previous 12 months were counted as sickness absenteeism (prevalence).

#### Data analysis and quality control

The collected data were checked and entered into Epi Info version 7 and analyzed by SPSS version 20. Descriptive analyses were computed by frequency distribution, mean, and percentages. All the variables with < 0.20 p-value in a bivariate analysis were exported to a multivariable logistic regression model to control the effects of potential confounders. The odds ratio (OR) with 95% confidence interval (CI) was used to test the strength of associations. A multi co-linearity assumption was checked using Variance Inflation Factor (VIF < 10). The significance of associations was established at ≤ 0.05 p-value. Goodness of fit (GoF) for a model was checked by Hosmer and Lemeshow test (> 0.05 p-value). To ensure the quality of data, 2 days training and orientation was provided for data collectors and supervisors. Before conducting the actual survey, a pilot test was performed on 5% (20 individuals) selected from Joy Tech Flower farm industry, which was not part of the final survey.

### Results

#### Socio-demographic characteristics

All of the sampled participants were interviewed (N = 444) with 100% response rate. More than half of the respondents, 55.6% (N = 247) were females. The mean age 24.2 (SD ± 6.6) was years. The majority, 66.9% (N = 297) belonged to the age group of 19–29 years. Regarding educational level, 42.1% (N = 187) had attended primary education, whereas 16% (N = 71) showed they had attended above secondary education (Table 1).

**Table 1:** Socio-demographic characteristics of participants in Bishoftu town, Ethiopia, 2018

Variables (N = 444)	Frequency (n)	Percentage (%)
Sex		
Male	197	44.4
Female	247	55.6
Age		
≤ 18	71	16.0
19–29	297	66.9
≥ 30	76	17.1
Marital status		
Single	249	56.1
Married	173	39.0
Divorced/widowed/separated	22	5.0
Educational status		
Cannot read and write	86	19.4
Primary education	187	42.1
Secondary education	100	22.5
Above secondary education	71	16.0
Monthly salary (in Birr)		
≤ 700	11	2.5
701–1500	385	86.7
> 1500	48	10.8
Work experience (in years)		
< 5	367	82.7
≥ 5	77	17.3
Type of employment		
Temporary	35	7.9
Permanent	409	92.1

*n* number

#### Prevalence and characteristics of sickness absenteeism

In this study, 54.5% (N = 242) [95% CI 50.2, 59.0] of the participants reported absent due to sickness of at least 3 consecutive working days in the past 12 months. A high proportion of sickness absence, 64% (n = 155) was indicated among women participants with a significant difference between the two groups (Person Chi square test (X^2^(1)) = 15.274; p-value <0.0001). In relation to marital status, 53% (n = 128) and 41.7% (n = 101) of the participants who manifested sickness absence spells indicated that they were single and married, respectively. The remaining 5.3% (n = 13) were divorced/widowed/separated. With regard to the working department, 55.4% (n = 134) of the respondents who indicated absenteeism as reasons for their illnesses were those who work in a greenhouse production department, 18.6% (n = 45) packing house, 10.3% (n = 25) spray room, 3.7% (n = 9) cold room, and 12% (n = 29) in the maintenance room. A total of 1357 working days were lost because of sickness absenteeism in the past 12 months (an average duration of 5.6 days per worker per year).

#### Reasons for sickness absenteeism

**Fig. 1:**
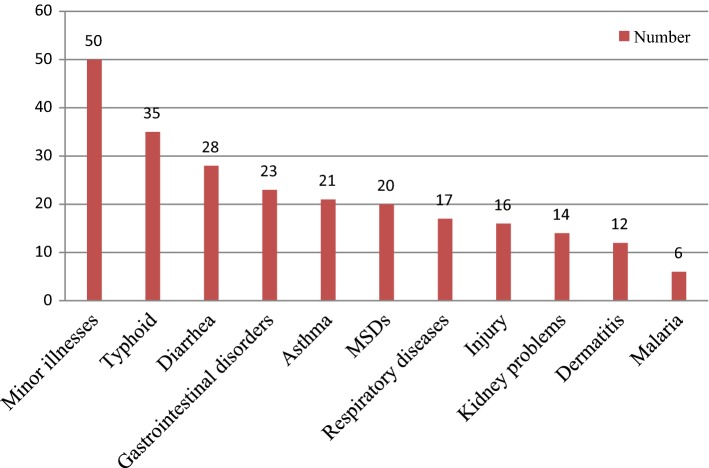
Reasons for sickness absenteeism among flower farm industry workers, Ethiopia, 2018 (n = 242)

The most common medical conditions that contributed to employees’ sickness absence were minor illnesses, 20.64% (n = 50). Among the sick absentees, 14.53% (n = 35) were due to typhoid diseases. Malaria, 2.48% (n = 6) was the least common cause of employees’ sickness absenteeism (Fig. 1).

#### Associated factors with sickness absence

In multivariable regression analysis, the factors which remained to considerably predict sickness absenteeism were, sex [AOR: 2.63; 95% CI (1.723, 4.036)], sickness presenteeism [AOR: 3.15; 95% CI (2.026, 4.904)], job satisfaction [AOR: 1.61; 95% CI (1.04, 2.34)] and drinking alcohol [AOR: 1.62; 95% CI (1.02, 2.59)].

The odds of sickness absenteeism were found to be 2.63 times more likely to occur among female participants as compared to male [AOR: 2.62; 95% CI (1.71, 4.01)]. The likely occurrence of sickness absenteeism was 3.15 times higher among study participants who sick and present at work than who do not [AOR: 3.16; 95% CI (2.03, 4.92)]. The chances of developing sickness absenteeism was 1.60 times higher among dissatisfied study participants than satisfied ones [AOR: 1.60; 95% CI (1.047, 2.462)] and the odds of sickness absenteeism is 1.64 times higher among the participants who drink alcohol than who do not drink [AOR: 1.64; 95% CI (1.023, 2.621)] (Table 2).

**Table 2:** Factors associated with sickness absenteeism in Bishoftu town, Ethiopia, 2018 (N = 444)

Variables	Sickness absenteeism		COR (95% CI)	AOR (95% CI)	P-value
	Yes	No			
Sex					
Female	155	92	2.13 (1.45, 3.12)	2.63 (1.723, 4.036)	0.0001**
Male	87	110	1	1	
Age group					
≤ 18 years	39	32	1	1	
19–29 years	153	144	0.87 (0.518, 1.466)	0.79 (0.449, 1.406)	0.430
≥ 30 years	50	26	1.57 (0.811, 3.070)	1.22 (0.557, 2.695)	0.613
Marital status					
Single	128	121	1.0	1.0	
Married	101	72	1.32 (0.897, 1.961)	1.17 (0.730, 1.902)	0.503
Divorced/widowed/separated	13	9	1.36 (0.563, 3.310)	1.31 (0.497, 3.485)	0.581
Educational status					
Cannot read and write	55	31	1.92 (1.130, 3.280)	1.48 (0.800, 2.755)	0.210
Primary education (1–8 grades)	105	82	1.39 (0.916, 2.108)	1.19 (0.743, 1.918)	0.464
Secondary and above education	82	89	1.0	1.0	
Monthly salary (in ETB Birr)					
≤ 700	9	2	3.80 (0.743, 19.51)	3.10 (0.515, 18.667)	0.217
701–1500	207	178	0.98 (0.539, 1.797)	0.69 (0.348, 1.394)	0.307
> 1500	26	22	1.0	1.0	
Work experience					
≥ 5 years	50	27	1.68 (1.013, 2.813)	1.02 (0.528, 2.001)	0.935
< 5 years	192	175	1	1	
Job satisfaction					
Satisfied	94	105	1.0	1.0	
Dissatisfied	148	97	1.70 (1.168, 2.488)	1.60 (1.047, 2.462)	0.003*
Work stress					
Not stressed	87	96	1.0	1.0	
Stressed	155	106	1.61 (1.102, 2.362)	1.46 (0.958, 2.237)	0.078
Periodic medical check up					
No	214	169	0.67 (0.390, 1.153)	0.65 (0.350, 1.221)	0.182
Yes	28	33	1.0	1.0	
Sickness presenteeism					
No	132	157	1.0	1.0	
Yes	110	45	2.90 (1.916, 4.412)	3.15 (2.026, 4.904)	0.0001**
Attendance incentive					
No	174	157	1.0	1.0	
Yes	68	45	1.36 (0.883, 2.105)	1.550 (0.963, 2.494)	0.071
Drinking alcohol					
No	163	151	1.0	1.0	
Yes	79	51	1.43 (0.947, 2.175)	1.64 (1.032, 2.621)	0.002*

*AOR* Adjusted odds ratio, *CI* confidence interval, *COR* crudes odds ratio, *ETB* Ethiopian Birr; *N* umber

*p-value < 0.05; **p-value < 0.001

p-value = 0.971 for Hosmer and Lemeshow test of model fitness

### Discussion

The prevalence of sickness absenteeism during the previous 12 months was 54.5% [(95% CI 50.2, 59.0)]. This finding was comparable with study conducted in Lume district, Ethiopia (58.8%) [33]. Similarities in the culture of reporting illnesses and injuries, socioeconomic level, and workplace illness management could be the possible reasons for these comparable results. The current finding was, however, higher than studies conducted in Nigeria (15.8–25.0%) [16, 17], India (18.6%) [11], and Brazil (31.5%) [15]. The difference might be due to methodological differences, study population, and variations due to disease patterns across the countries.

In this study, multivariable analysis indicated that sex is a significant predictor of sickness absenteeism. This result replicated findings of previous studies [4, 18, 36]. This could be due to the fact that the way different health conditions perceived by women and men differ. Women also usually engage in multiple roles (work/home interface). This in turn probably worsens their conditions due to lack of the necessary rests and eventually leads them to be away off their paid duties. The other study has provided similar explanation [21]. Further, currently, the labor markets are usually segregated into male and female occupations. This could suggest that absence due to ill health can be explained by the gender compositions of the workplaces. The situation was practical in the present study in that the proportions of females predominately observed than male workers. This had extended the previous explanation [37].

We found sickness presenteeism was the factor that markedly affected employees’ absence from work. This was supported by previous studies [38, 39]. The plausible reason for this result was due to that working while sick may exacerbate workers’ health conditions resulting in subsequent repeated away off work because of lack of the necessary recuperation. Moreover, this also possibly suggest that difficulties in pressuring oneself to work when one is not feeling well can lead to not performing effectively, and this ultimately leads to a risk of increased frequencies of sickness absenteeism. Other investigators have also provided equal explanations [38, 40].

The multivariable logistic regression analysis revealed significant associations of job satisfaction and sickness absenteeism. This was in concordant with other findings [23, 29, 36]. A job dissatisfied workers might often think differently than a job satisfied workers. The concomitant employee’s health condition (the health condition under the study) and dissatisfaction with a job could predispose workers to feel unhappy resulting in later absence from their work.

### Conclusions

Sickness absenteeism had been found common in this study. Sex, job satisfaction, sickness presenteeism, and alcohol consumption were the factors associated with sickness absenteeism. Therefore, employers and policy designers need to formulate preventive schemes focusing on gender difference, job satisfaction, and concomitant tackling of sickness absenteeism and presenteeism. Workplace programs targeting to health risk behaviors, like alcohol consumption is also imperative to curb the problem.

### Strength and limitations

One of the strengths of the current study was that the data generated were supported by the industry registered sickness absence. This could substantiate the validity of the data collected and minimizes the potential recall bias anticipated. On the other hand, a few drawbacks could be observed in the present study. One thing was that the samples for this study were drawn only from the specific industry (flower farm industry). Therefore, it might be uncertain to conclude the findings for the other industries. Future studies would better focus on allocating a large sample from varieties of industry sectors with a longitudinal study design.

## Abbreviations

AOR: adjusted odds ratios
BMI: body mass index
BSc: Bachelor of Science
CI: confidence interval
COR: crudes odds ratios
KM: kilometer
MPH: masters of public health
OR: odds ratios
SD: standard deviation
SPSS: Statistical Package for Social Sciences
